# Measuring what matters: evaluating the psychometric properties of goal attainment scaling in a psychosocial dementia trial

**DOI:** 10.1093/ageing/afag260

**Published:** 2026-09-14

**Authors:** Jessica Budgett, Harriet Demnitz-King, Andrew Sommerlad, Nuriye Kupeli, Victoria Vickerstaff, Claudia Cooper

**Affiliations:** Centre for Psychiatry and Mental Health, Wolfson Institute of Population Health, Queen Mary University of London, London, England, UK; Division of Psychiatry, University College London, London, England, UK; Centre for Psychiatry and Mental Health, Wolfson Institute of Population Health, Queen Mary University of London, London, England, UK; Division of Psychiatry, University College London, London, England, UK; Centre for Psychiatry and Mental Health, Wolfson Institute of Population Health, Queen Mary University of London, London, England, UK; Division of Psychiatry, University College London, London, England, UK; Centre for Evaluation and Methods, Wolfson Institute of Population Health, Queen Mary University of London, London, England, UK; Centre for Psychiatry and Mental Health, Wolfson Institute of Population Health, Queen Mary University of London, London, England, UK

**Keywords:** Goal Attainment Scaling, psychometrics, quality of life, psychosocial intervention, dementia care, older people

## Abstract

**Background:**

It can be challenging to identify clinically meaningful, responsive primary outcomes for psychosocial intervention trials in people with dementia. Goal Attainment Scaling (GAS), a personalised, patient-generated outcome measure, enables individuals to define and evaluate progress toward meaningful goals. Evidence regarding its psychometric properties in dementia trials, however, remains limited.

**Objective:**

To evaluate the feasibility, reliability, and validity of GAS within the randomised controlled trial of NIDUS-Family (New Interventions in Dementia Study), a goal-based psychosocial intervention.

**Methods:**

302 people with dementia and family carer dyads collaboratively set baseline goals toward supporting the person with dementia to live well at home. In a psychometric evaluation guided by the COnsensus-based Standards for the selection of health Measurement INstruments (COSMIN) framework, we examined follow-up completion (measuring feasibility); rater agreement; intra-rater reliability; construct validity by assessing correlations between change in GAS and quality-of-life (DEMQOL-proxy and CarerQoL); and predictive validity by testing whether GAS change predicted living at home at 12 months.

**Results:**

GAS completion rates were 86% at 6 months and 82% at 12 months. GAS demonstrated excellent inter-rater (Intraclass Correlation Coefficient = 0.99) and intra-rater reliability (Cohen’s kappa = 0.91). There were significant but weak positive correlations between GAS change and carer and person with dementia quality-of-life (r = 0.14–0.22). GAS change was associated with remaining at home (odds ratio 1.08 per point increase; *P* <.01).

**Conclusion:**

GAS is feasible, valid and reliable, so may be a sensitive and clinically meaningful candidate primary outcome for psychosocial dementia trials. Correlations with global quality of life were significant but modest, consistent with GAS capturing personalised rather than generic outcomes.

## Key points

- This is the largest psychometric evaluation of Goal Attainment Scaling (GAS) in a non-pharmacological dementia trial.
- GAS showed excellent reliability and high follow-up completion.
- Weak correlations with global quality-of-life measures suggest GAS captures personalised rather than generic outcomes.
- Greater goal attainment was associated with increased odds of remaining at home at 12 months.
- GAS offers a feasible and scalable personalised outcome measure for dementia trials.

## Introduction

An estimated 982 000 people live with dementia in the UK [1]. Most wish to remain living at home for as long as possible [2]. Dementia is a heterogeneous condition, for which it can be challenging to identify clinically meaningful, responsive trial primary outcome measures, especially when evaluating multicomponent psychosocial interventions.

Goal Attainment Scaling (GAS) is a personalised outcome measure. Individuals are supported to define a goal and describe their ‘baseline’ performance on this goal, and benchmark measures of what significant (a level scored as +1) and very significant (+2) progress, and deterioration (−1 and −2) on each goal would look like; this provides a personalised scale to evaluate goal progress. GAS can capture individualised change not reflected in standardised instruments [3]. Evidence for GAS psychometric properties in dementia trials remains limited [3], partly because it assesses individualised constructs with variable content and number across participants [4].

The NIDUS-Family (New Interventions in Dementia Study) randomised controlled trial (RCT) evaluated a fully manualised, modular psychosocial intervention comprising 6–8 support sessions delivered by trained non-clinical facilitators, tailored to goals that dyads (family carers, with care recipients with dementia where possible) set [5].

GAS, the primary outcome, demonstrated a significant intervention effect at 12 [6] and 24 months [7]. A previous content analysis categorised the 1043 baseline individualised goals into nine domains: five focused on the person living with dementia (mood, behaviour, self-care, cognition and instrumental activities of daily living) and four on family carers (carer mood, carer break, carer behaviour and carer sleep) [8]. The findings demonstrated content validity and the feasibility of nonclinical facilitators supporting goal-setting in this population: they enabled 313/328 (95%) participants to collaboratively set three to five goals.

It is less clear how GAS performs psychometrically as a longitudinal outcome within a dementia RCT. This study evaluates the feasibility, reliability and validity of GAS at 6 and 12 months in people living with dementia and family carers. We hypothesised *a priori* that GAS would demonstrate high feasibility and reliability, weak-to-moderate associations with quality-of-life measures (reflecting related but distinct constructs) and show predictive validity for remaining at home.

## Methods

This study is a psychometric evaluation of GAS data collected within the NIDUS-Family multi-site, parallel-group RCT conducted across 21 sites in England between March 2020 and May 2022 (ISRCTN11425138). The trial received ethics approval from Camden & Kings Cross Research Ethics Committee (19/LO/1667). Analyses used baseline, 6 months and 12-month data. Trial effectiveness findings are reported elsewhere [6].

Participant dyads were community-dwelling people living with dementia (any subtype or severity) and an English-speaking family carer with at least weekly contact, recruited via NHS memory clinics, older adult mental health services and primary care. In total, 302 dyads were randomised. Dyads provided informed consent/consultee agreement [5]*.*

At baseline, trained non-clinical facilitators supported dyads to set three to five goals focused on living as well as possible at home. Each goal was defined across five attainment levels (−2 to +2) with specific descriptors. The full outcome assessment battery is reported elsewhere [6]. Living situation was assessed using the Client Service Receipt Inventory, operationalised in this analysis as a binary outcome: living at home versus not living at home, the latter including care-home residence or death (whether or not living in a residential care facility) [9].

At 6- and 12-months, facilitators invited all participants to describe the current situation and assess goal attainment against the baseline-defined scale levels. Goals were rated by the family carer (and person living with dementia, where possible) and by the facilitator, and both scores were recorded.

With consent, 30 12-month goal-scoring discussions were audio- or video-recorded. From these, 102 goal-scoring discussions (edited to retain the discussion only, excluding the original attainment rating) were re-scored by the same facilitator (three facilitators took part) after 8–9 weeks.

### Data analysis

Psychometric evaluation was guided by an adapted version of the COnsensus-based Standards for the selection of health Measurement Instruments (COSMIN) framework for GAS in clinical trials recommended by Gaasterland’s (2019) [4] [10](Appendix S1 in Supplementary Data section).

We calculated standardised GAS T-scores at 6 and 12 months using the Kiresuk–Sherman formula [11] (assuming inter-goal correlation ρ = 0.3), where T = 50 represents no change from baseline and higher scores indicate improvement. Feasibility was assessed using completion rates; ≥75% was considered acceptable [12, 13].

Agreement between facilitator and carer/dyad GAS T scores at 6 and 12 months was examined using intraclass correlation coefficients (ICCs) (two-way random-effects model, absolute agreement). Intra-rater reliability was evaluated by comparing the two ratings completed for each of the 30 recordings. Agreement on the five-point attainment ratings was quantified using weighted Cohen’s kappa, with values ≥0.60 considered indicative of substantial agreement [14].

Construct validity was evaluated using Pearson correlations between GAS T-scores and change in DEMQOL-proxy (person living with dementia’s quality of life) and CarerQoL [14] (carer-related quality of life) from baseline to 6 and 12 months. Based on prior content analysis [8], goals were categorised as person with dementia-focused or carer-focused, and correlations were repeated within these subsets.

Predictive validity was assessed using univariable logistic regression examining whether 12-month GAS T-scores were associated with living situation at 12 months, defined as a binary outcome: living at home versus not living at home, the latter including care-home residence or death. 114 participants with unknown living status were excluded from this analysis.

All analyses were conducted in RStudio (version 4.4.2) using the psych and dplyr packages. Results are presented with 95% confidence intervals and two-sided *P*-values (α = 0.05).

## Results

Six-month assessments occurred at a median of 191 days (IQR 181–204) and 12-month assessments at 374 days (IQR 364–391) after baseline. See Appendix S2 in the Supplementary Data section for completion rates and outcome scores; socio-demographic characteristics are in Appendix S3.

### Feasibility

261/302 dyads (86%) completed GAS at 6 months and 247 (82%) at 12 months, both exceeding the pre-specified feasibility threshold of 75%.

### Reliability

Agreement between facilitator- and carer/dyad-rated GAS T-scores was strong. The ICC was 0.99 at both 6 (*n* = 261) and 12 months (*n* = 247) (95% CI 0.99–0.99, *P* <.001).

The goal-scoring discussions (30 participants, 102 goals) were re-scored by facilitator after a mean interval of 60 days (SD = 3, range 55–65 days). Weighted Cohen’s kappa for repeated scoring was 0.91 (z = 13.4, *P* <.001), demonstrating strong intra-rater reliability.

### Construct validity

Table 1 summarises the Pearson correlation coefficients between GAS T-scores and change in quality-of-life measures. Across analyses, correlation coefficients were consistently small (r = 0.11–0.25).

**Table 1:** Summary of the Peason correlations between DEMQOL, CarerQoL and GAS-T scores.

	DEMQOL-proxy change score, 6 months	DEMQOL-proxy change score, 12 months	CarerQoL change score, 6 months	CarerQoL change score, 12 months
GAS T scores (All goals)	r = 0.20, CI 0.06, 0.35, *P* = .005 *n* = 205	r = 0.15, CI -0.01, 0.30 *P* = .06 *n* = 161	r = 0.14, CI 0.001, 0.27 *P* = .049 *n* = 200	r = 0.22, CI 0.07, 0.37 *P* = .005 *n* = 158
GAS T scores (PLWD goals)	r = 0.23, CI 0.09, 0.35, *P* = .001 *n* = 203	r = 0.07, CI 0.08, 0.23 *P* = .351 *n* = 160		
GAS T scores (Carer goals)			r = 0.11, CI -0.05, 0.27 *P* = .184 *n* = 150	r = 0.25, CI 0.07, 0.41 *P* = .006 *n* = 123

Abbreviations: *r* = Pearson correlation coefficient; CI, confidence interval; *P* = significance level; *n* = number of dyads included in each correlation analysis.

### Predictive validity

**Figure 1:**
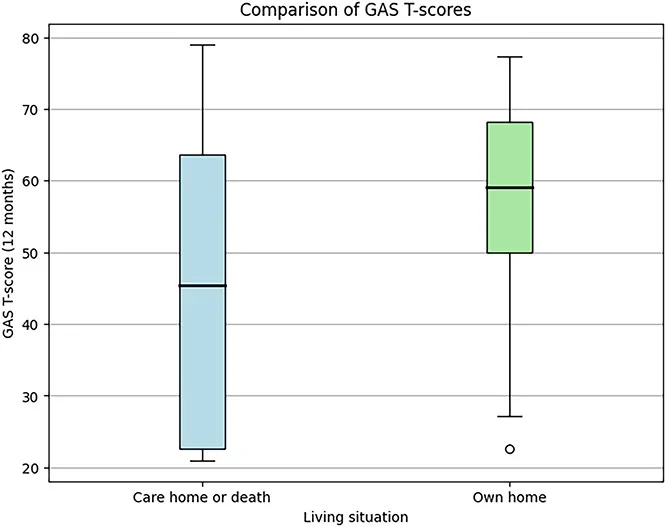
Logistic regression model: distribution of GAS T-scores by living situation at 12 months (own home vs. care home or death), excluding participants with unknown living situation.

At 12 months, 139 (46%) participants were living at home, 29 (10%) in care homes, and 20 (7%) had died; living status was unknown for the remainder (*n* = 114). Logistic regression demonstrated that a one-point increase in GAS change score was associated with increased odds (odds ratio 1.08 (95% CI, 1.04–1.11)) for remaining at home rather than entering residential care or dying (Figure 1).

## Discussion

This is the first large-scale psychometric evaluation of GAS within a psychosocial dementia RCT. We found that GAS demonstrated follow-up feasibility, strong agreement between raters, strong intra-rater reliability, weak evidence of construct validity, and predictive validity in relation to a clinically meaningful outcome. Despite its personalised and non-standardised nature, these results suggest that with structured training and supervision, GAS can be implemented consistently.

Associations between GAS and quality-of-life measures were weak but significant. COSMIN-based frameworks anticipate modest associations when instruments assess related, non-identical constructs [10].

GAS captures progress in domains defined by individual priorities, whereas standardised instruments assess broader wellbeing or functioning. Our findings suggest GAS can make a useful contribution to research and practice when used alongside, rather than instead of conventional outcome measures, by capturing what matters most to individuals living with dementia. Notably, in the main NIDUS-Family RCT and another psychosocial dementia trial, goal attainment improved despite minimal change on conventional measures [6, 15].

Higher GAS scores were associated with an increased likelihood of remaining at home at 12 months, suggesting that personalised goals attainment may relate to meaningful real-world outcomes. Continued independent living is a key outcome in dementia care [16] and health policy frameworks [17].

GAS aggregates heterogeneous and multidimensional goals, challenging assumptions of one-dimensionality underlying many psychometric frameworks. Evaluating its properties using conventional COSMIN criteria is therefore complex. We applied an adapted framework [4] to assess reliability and validity; however, some properties expected of fixed-item measures (e.g. internal consistency) do not directly apply to personalised goal-based tools. Construct validation was restricted to global quality-of-life measures, most of which were proxy-reported. Predictive analyses were based on complete cases and may not generalise to participants with missing follow-up data; missingness may have been associated with a lesser likelihood of remaining at home.

There remains a need for improved reporting standards in GAS research. Currently, studies vary widely in how they describe GAS methodology and approach psychometric analysis [3, 18, 19]. A consensus-based reporting guideline would enhance transparency and comparability across populations and settings.

Overall, these findings support GAS as a feasible, reliable and clinically meaningful personalised outcome measure in dementia trials. As health systems increasingly emphasise person-centred care [17, 20–22], evaluation of personalised measures such as GAS will be essential to capturing outcomes that reflect what matters most to people living with dementia and their families.

## Supplementary Material

Supplementary_materials_afag260

## Data Availability

The data underlying this article will be shared on reasonable request to the corresponding author, subject to approval by the NIDUS trial study team and relevant governance processes.
