# Supplementary material for: Measuring what matters: evaluating the psychometric properties of goal attainment scaling in a psychosocial dementia trial
Source: Age Ageing. 2026 Sep 14;55(9):afag260. doi: 10.1093/ageing/afag260 (PMC13574283; doi:10.1093/ageing/afag260)
Supplement: Supplementary file 1 [file supplementary_materials_afag260.docx]

**Measuring what matters: evaluating the psychometric properties of goal attainment scaling in a psychosocial dementia trial**

**Supplementary materials**

***Appendix 1: Adapted COSMIN (COnsensus-based Standards for the selection of health Measurement INstruments) Risk of Bias boxes***

| ***CONTENT VALIDITY*** | ***Very Good*** | ***Adequate*** | ***Doubtful*** | ***Inadequate*** | ***Not applicable*** |
| --- | --- | --- | --- | --- | --- |
| *1.Were the goals set reviewed or evaluated by one or more independent experts?*  *Experts can be considered broadly and may include clinicians, patients, or family members****.***  ***(Gaasterland et al., 2019)[31]*** | *The goals set were reviewed or evaluated by one or more independent experts and the method in which this was done is clearly stated.* | *Assumable that the goals set were reviewed or evaluated by one or more independent experts.* | *Not clear if goals set were reviewed or evaluated by one or more independent expert or there were no reviewers of the goals set.* | *** |  |
| *2.Were the goal achievement levels/scale evaluated by independent clinical experts.*  ***(Gaasterland et al., 2019)[31]*** | *The goal achievement levels/scale was evaluated by independent clinical experts and the method in which this was done is clearly stated.* | *Assumable that the goal achievement levels/scale was evaluated by independent clinical experts* | *Not clear if the goal achievement levels/scale was evaluated by independent clinical experts or there were no independent evaluators.* | *** |  |
| *3.Was the target population (PLWD or FCs) involved in goal selection/setting?*  ***(Gaasterland et al., 2016)[60]*** | *The target population was involved in the goal selection/ setting* | *Assumable that the target population was involved OR they were not involved but a suitable explanation to why not is provided* | *Not clear if the target population was involved in the goal selection/ setting or they were not involved, and no explanation provided* | **** |  |
| *4.Were goals formulated according to the criteria ‘Specific, Measurable, Attainable, Realistic, and Time-bound’ (SMART)*  ***Gaasterland et al. (2016)[60]*** | *The SMART criteria was checked by second reviewer.* | *Some elements of SMART criteria or similar were applied to goal setting but it may not mention whether this was checked by an independent evaluator.* | *Not clear if any SMART criteria was applied to the goals.* | *** |  |
| *5.Were the goal areas summarised or coded into goal areas or goal domains? Evidence of content analysis of identified goal areas.*  ***(Shankar et al., 2020) [64]***  ***(Bouwens et al., 2008)[41]*** | *Clear evidence of some form of content analysis of identified goal areas – the results of this is provided.* | *Some evidence of some form of content analysis of identified goal areas – but results may not be presented in the paper.* | *Not clear if any content analysis of goal areas was done.* | *** |  |
| *6.Were skilled facilitators used or were facilitators/raters suitably trained?*  ***(Adapted from COMSIN [62])*** | *Evidence of facilitators receiving suitable amount of training (mentions training sessions or role play practice).*  *OR clear that facilitators were already sufficiently skilled.* | *Facilitators had limited training or experience in using goal outcome measure.*  *Or facilitators had limited experience in the dementia field* | *Not clear if facilitators were trained or not trained.* | *Evidence that facilitators were not appropriately skilled* |  |

*^Abbreviations: PLWD, People Living with Dementia; FCs, Family carers; SMART (specific, measurable, attainable, realistic, and time-bound)^*

*^*Item is considered ideal or recommended to evaluate content validity but it is not an essential or well described process of the GAS method and therefore we do not require the study to be listed as inadequate if it does not do this but listed as doubtful instead.^*

*^** Having the target population involved in the setting or selection of goals is ideal but is not an essential part of GAS and in some studies valid explanations of why they were not included are provided.^*

| ***CONSTRUCT VALIDITY***  ***(Trial Level, hypothesis testing)*** | ***Very Good*** | ***Adequate*** | ***Doubtful*** | ***Inadequate*** | ***Not applicable*** |
| --- | --- | --- | --- | --- | --- |
| *1.Were specific hypotheses formulated?*  ***(Gaasterland et al., 2019)[31]*** | *Adequate*  *description of the*  *hypotheses* |  | *Poor description of*  *the hypotheses* | *NO description of*  *the hypotheses* |  |
| *2.Was an adequate description provided of the intervention given?*  ***(COSMIN Box 10.d [62])*** | *Adequate*  *description of the*  *intervention* |  | *Poor description of*  *the intervention* | *NO description of*  *the intervention* |  |
| *3.Was the statistical method appropriate for the hypotheses to be tested?*  ***(COSMIN, Box 10d [62])*** | *Statistical method*  *was appropriate* | *Assumable that*  *statistical method*  *were appropriate* | *Statistical method*  *applied NOT optimal* | *Statistical method*  *applied NOT*  *appropriate* |  |
| *4.Were there any other important flaws in the design or statistical methods of the study?*  *(****COSMIN, Box 10.d [62])*** | *No other important*  *methodological*  *flaws* |  | *Other minor*  *methodological flaws* | *Other important*  *methodological*  *flaws* |  |
| ***CONSTRUCT VALIDITY (Individual level / Comparison with secondary measure)*** | ***Very Good*** | ***Adequate*** | ***Doubtful*** | ***Inadequate*** | ***Not applicable*** |
| *1.Is it clear what the comparator instrument(s) measure?*  ***(COSMIN, Box 10b [62])*** | *Constructs*  *measured by the*  *comparator*  *instrument(s) is*  *clear* |  |  | *Constructs*  *measured by the*  *comparator*  *instrument(s) is*  *not clear* |  |
| *2.Were the measurement properties of the comparator instrument(s) sufficient?*  ***(COSMIN, box 10b [62])*** | *Sufficient*  *measurement*  *properties of the*  *comparator*  *instrument(s) in a*  *population similar to*  *the study population* | *Sufficient*  *measurement*  *properties of the*  *comparator*  *instrument(s) but*  *not sure if these*  *apply to the study*  *population* | *Some information on*  *measurement*  *properties of the*  *comparator*  *instrument(s) in any*  *study population* | *NO information on*  *the measurement*  *properties of the*  *comparator*  *instrument(s) OR*  *evidence of poor*  *quality of*  *comparator*  *instrument(s)* |  |
| *3.Was the statistical method appropriate for the correlations to be tested?*  ***(COSMIN, box 10b [62])*** | *Statistical method*  *was appropriate* | *Assumable that*  *statistical method*  *were appropriate* | *Statistical method*  *applied NOT optimal* | *Statistical method*  *applied NOT*  *appropriate* |  |

| ***RELIABILITY (COSMIN, box 6 [62])*** | ***Very Good*** | ***Adequate*** | ***Doubtful*** | ***Inadequate*** | ***Not applicable*** |
| --- | --- | --- | --- | --- | --- |
| *1.Were the test conditions similar for the measurements? E.g type of administration, environment, instructions* | *Test conditions were similar (evidence provided)* | *Assumable that test conditions were similar* | *Unclear if test conditions were similar* | *Test conditions were NOT similar* |  |
| *2.Was the time interval appropriate?* | *Time interval appropriate* |  | *Doubtful whether time interval was appropriate or time interval was not stated* | *Time interval NOT appropriate* |  |
| *3.For continuous scores: Was an intraclass correlation coefficient (ICC) calculated?* | *ICC calculated and model or formula of the ICC is described* | *ICC calculated but model or formula of the ICC not described or not optimal. Pearson or Spearman correlation coefficient calculated with evidence provided that no systematic change has occurred* | *Pearson or Spearman correlation coefficient calculated WITHOUT evidence provided that no systematic change has occurred or WITH evidence that systematic change has occurred* | *No ICC or Pearson or Spearman correlations calculated* | *Not applicable* |
| *4.For dichotomous/nominal/ordinal scores: Was kappa calculated?* | *Kappa calculated* |  |  | *No kappa calculated* | *Not applicable* |
| *5.For ordinal scores: Was a weighted kappa calculated?* | *Weighted Kappa calculated* |  | *Unweighted Kappa calculated or not described* |  | *Not applicable* |
| *6.For ordinal scores: Was the weighting scheme described? e.g. linear, quadratic* | *Weighting scheme described* | *Weighting scheme NOT described* |  |  | *Not applicable* |
| *7.Were there any other important flaws in the design or statistical methods of the study?* | *No other important methodological flaws* |  | *Other minor methodological flaws* | *Other important methodological flaws* |  |

|  | **Visit** | | | | | | | | | | | | | | | | | | | | | |  | | |  | | | |  | | |
| --- | --- | --- | --- | --- | --- | --- | --- | --- | --- | --- | --- | --- | --- | --- | --- | --- | --- | --- | --- | --- | --- | --- | --- | --- | --- | --- | --- | --- | --- | --- | --- | --- |
|  | **Baseline** | | | | | **6 Months** | | | | | | | | | | | | **12 Months** | | | | | | | | | | | | | | |
|  | **n** | **Mean** | | **SD** | | **n** | **Mean** | | **SD** | | **Min** | | **Med** | | **Max** | | | **n** | **Mean** | | **SD** | | | **Min** | | | **Med** | | **Max** | | |  |
| **GAS_participant*** | n/a | |  | |  | 261 | | 57.0 | | (12.2) | | 21.0 | | 59.1 | | 77.4 | 247 | | | 54.5 | | (14.9) | | | 21.0 | | | 55.3 | | | 79.0 | |
| **GAS_researcher**** | n/a | |  | |  | 261 | | 56.8 | | (12.2) | | 21.0 | | 59.1 | | 77.4 | 247 | | | 54.5 | | (15.0) | | | 21.0 | | | 56.0 | | | 79.0 | |
| **DEMQOL-proxy** | 301 | | 89.5 | | (14.6) | 205 | | 94.1 | | (13.4) | | 53.0 | | 95.0 | | 121.0 | 162 | | | 93.2 | | (14.8) | | | 41.0 | | | 95.0 | | | 120.0 | |
| **CarerQoL** | 300 | | 76.0 | | (16.9) | 202 | | 79.1 | | (15.6) | | 21.6 | | 82.3 | | 100.0 | 158 | | | 79.6 | | (13.7) | | | 31.6 | | | 82.2 | | | 100.0 | |
| **CSRI (Q. 3)***** | 300 | |  | |  | 207 | |  | |  | |  | |  | |  | 168 | | |  | |  | | |  | | |  | | |  | |

**Appendix 2:** *Summary of the number of completed outcome measures across the 12-month RCT, including the mean, standard deviation, minimum, median and maximum score.*

*Abbreviations: GAS=Goal Attainment Scaling, n=number completed, SD=standard deviation, Min=minimum score, Med=median score, Max=maximum score, DEMQOL-proxy=Dementia Quality of Life measure (proxy rated), CarerQo=Care-Related Quality of Life Instrument, CSRI=Client Service Receipt Inventory*

**GAS scored by the family carer or participating dyad*

***GAS scores by the researcher facilitator*

****CSRI question 3: Has your relative with dementia been admitted to a care home or other supported residential accommodation in the* past 6 months?

***Appendix 3: Baseline characteristics of PLWD and family carers***

|  | ***PLWD*** | | ***Family carers*** | |
| --- | --- | --- | --- | --- |
| *Results are n^*^ (%) unless specified otherwise* | ***N=302*** | | ***N=302*** | |
| ***Age*** *(years), mean (SD^†^)* | *79.9* | *(8.2)* | *63.4* | *(12.5)* |
| ***Ethnicity*** |  |  |  |  |
| *White* | *266* | *(88.1)* | *266* | *(88.1)* |
| *Mixed* | *4* | *(1.3)* | *4* | *(1.3)* |
| *Asian* | *17* | *(5.6)* | *16* | *(5.3)* |
| *Black* | *11* | *(3.6)* | *11* | *(3.6)* |
| *Other* | *4* | *(1.3)* | *5* | *(1.7)* |
| ***First language*** |  |  |  |  |
| *English* | *260* | *(86.1)* | *277* | *(91.7)* |
| *Other* | *42* | *(13.9)* | *25* | *(8.3)* |
| ***Gender*** |  |  |  |  |
| *Male* | *133* | *(44.0)* | *90* | *(29.8)* |
| *Female* | *169* | *(56.0)* | *212* | *(70.2)* |
| ***Marital status*** |  |  |  |  |
| *In relationship* | *178* | *(58.9)* | *251* | *(83.1)* |
| *Divorced* | *17* | *(5.6)* | *11* | *(3.6)* |
| *Single* | *7* | *(2.3)* | *35* | *(11.5)* |
| *Widowed* | *100* | *(33.1)* | *5* | *(1.7)* |
| ***Highest educational attainment*** |  |  |  |  |
| *Higher degree* | *33* | *(11.1)* | *55* | *(18.2)* |
| *Degree* | *56* | *(18.9)* | *97* | *(32.1)* |
| *A level (or equivalent)* | *58* | *(19.2)* | *80* | *(26.5)* |
| *GCSE (or equivalent)* | *102* | *(33.8)* | *55* | *(18.2)* |
| *No formal qualifications* | *47* | *(15.9)* | *15* | *(5.0)* |
| ***Living situation*** |  |  |  |  |
| *Live alone* | *84* | *(27.8)* | *153* | *(50.7)* |
| *Live with partner/spouse* | *159* | *(52.6)* | *137* | *(45.4)* |
| *Live with children* | *39* | *(12.9)* | *1* | *(0.3)* |
| *Other* | *20* | *(6.6)* | *11* | *(3.6)* |
| ***Co-habiting*** |  |  |  |  |
| *Dyad are co-habiting* | *193* | *(63.9)* |  |  |
| *Dyad live apart* | *109* | *(36.1)* |  |  |
| ***Accommodation*** |  |  |  |  |
| *Council rented* | *20* | *(6.6)* | *13* | *(4.3)* |
| *Housing association rented* | *14* | *(4.6)* | *217* | *(71.9)* |
| *Private rented* | *31* | *(10.3)* | *12* | *(4.9)* |
| *Owner-occupied* | *237* | *(78.5)* | *27* | *(8.9)* |
| ***Carer relationship*** |  |  |  |  |
| *Wife / partner* |  |  | *99* | *(32.8)* |
| *Husband / partner* |  |  | *52* | *(17.2)* |
| *Daughter* |  |  | *109* | *(36.1)* |
| *Son* |  |  | *35* | *(11.6)* |
| *Other* |  |  | *7* | *(2.3)* |
| ***Dementia diagnosis*** |  |  |  |  |
| *Alzheimer’s Disease* | *139* | *(46.0)* |  |  |
| *Vascular dementia* | *38* | *(12.6)* |  |  |
| *Lewy body dementia* | *10* | *(3.3)* |  |  |
| *Frontotemporal dementia* | *8* | *(2.6)* |  |  |
| *Other* | *84* | *(27.8)* |  |  |
| *Unable to specify* | *23* | *(7.6)* |  |  |

*^*^N = total number of participants; ^†^SD = standard deviation*
